# Temporal Association of Certain Neuropsychiatric Disorders Following Vaccination of Children and Adolescents: A Pilot Case–Control Study

**DOI:** 10.3389/fpsyt.2017.00003

**Published:** 2017-01-19

**Authors:** Douglas L. Leslie, Robert A. Kobre, Brian J. Richmand, Selin Aktan Guloksuz, James F. Leckman

**Affiliations:** ^1^Department of Public Health Sciences, Pennsylvania State University College of Medicine, Hershey, PA, USA; ^2^Yale Child Study Center, Yale University School of Medicine, New Haven, CT, USA

**Keywords:** anorexia nervosa, obsessive–compulsive disorder, anxiety disorder, tic disorder, vaccination, influenza, meningococcus

## Abstract

**Background:**

Although the association of the measles, mumps, and rubella vaccine with autism spectrum disorder has been convincingly disproven, the onset of certain brain-related autoimmune and inflammatory disorders has been found to be temporally associated with the antecedent administration of various vaccines. This study examines whether antecedent vaccinations are associated with increased incidence of obsessive–compulsive disorder (OCD), anorexia nervosa (AN), anxiety disorder, chronic tic disorder, attention deficit hyperactivity disorder, major depressive disorder, and bipolar disorder in a national sample of privately insured children.

**Methods:**

Using claims data, we compared the prior year’s occurrence of vaccinations in children and adolescents aged 6–15 years with the above neuropsychiatric disorders that were newly diagnosed between January 2002 and December 2007, as well as two control conditions, broken bones and open wounds. Subjects were matched with controls according to age, gender, geographical area, and seasonality. Conditional logistic regression models were used to determine the association of prior vaccinations with each condition.

**Results:**

Subjects with newly diagnosed AN were more likely than controls to have had any vaccination in the previous 3 months [hazard ratio (HR) 1.80, 95% confidence interval 1.21–2.68]. Influenza vaccinations during the prior 3, 6, and 12 months were also associated with incident diagnoses of AN, OCD, and an anxiety disorder. Several other associations were also significant with HRs greater than 1.40 (hepatitis A with OCD and AN; hepatitis B with AN; and meningitis with AN and chronic tic disorder).

**Conclusion:**

This pilot epidemiologic analysis implies that the onset of some neuropsychiatric disorders may be temporally related to prior vaccinations in a subset of individuals. These findings warrant further investigation, but do not prove a causal role of antecedent infections or vaccinations in the pathoetiology of these conditions. Given the modest magnitude of these findings in contrast to the clear public health benefits of the timely administration of vaccines in preventing mortality and morbidity in childhood infectious diseases, we encourage families to maintain vaccination schedules according to CDC guidelines.

## Introduction

There is a considerable body of scientific evidence indicating that the immune system plays a key role in normal brain development and in the pathobiology of several neuropsychiatric disorders (1). These include obsessive–compulsive disorder (OCD) (2, 3), anorexia nervosa (AN) (4), tic disorders (5), attention deficit hyperactivity disorder (ADHD) (6), major depressive disorder (7), and bipolar disorder (8). The precise role immune mechanisms play in these disorders remains to be determined.

In light of the role of the immune system in these central nervous system (CNS) conditions, the impact of vaccines on childhood-onset neuropsychiatric diseases had been considered and was mainly addressed with regards to the administration of the measles, mumps, and rubella (MMR) vaccine (and its various components) and the subsequent development of autism spectrum disorder (ASD). Although the controversy over MMR vaccination and ASD still exists for some members of the public, this association has been convincingly disproven (9, 10). On the other hand, the onset of a limited number of autoimmune and inflammatory disorders affecting the CNS has been found to be temporally associated with the antecedent administration of various vaccines (11). These disorders include idiopathic thrombocytopenic purpura, acute disseminated encephalomyelitis, and Guillain–Barré syndrome among others (12–16). More recently, data have emerged indicating an association between the administration of the H1N1 influenza vaccine containing the AS03 adjuvant and the subsequent new onset of narcolepsy in several northern European countries (17, 18). The immune mechanisms and host factors underlying these associations have not been identified or fully characterized, although preliminary data are beginning to emerge (18–23).

Given this growing body of evidence of immunological involvement in CNS conditions, and despite the controversy concerning the link between ASD and MMR and the clear public health importance of vaccinations, we hypothesized that some vaccines could have an impact in a subset of susceptible individuals and aimed to investigate whether there is a temporal association between the antecedent administration of vaccines and the onset of several neuropsychiatric disorders, including OCD, AN, tic disorder, anxiety disorder, ADHD, major depressive disorder, and bipolar disorder using a case–control population-based pediatric sample (children aged 6–15 years). To assess the specificity of any statistical associations, we also determined whether or not there were any temporal associations between antecedent vaccine administration and the occurrence of broken bones or open wounds.

## Materials and Methods

Data were obtained from the MarketScan^®^ Commercial Claims and Encounters database, which is constructed and maintained by Truven Health Analytics. Data from 2002 to 2007 were used for the study. MarketScan consists of de-identified reimbursed health-care claims for employees, retirees, and their dependents of over 250 medium and large employers and health plans. Hence, individuals included in the database are covered under private insurance plans; no Medicaid or Medicare data are included. The database includes claims information describing the health-care experiences for approximately 56 million covered lives per year. The database is divided into subsections, including inpatient claims, outpatient claims, outpatient prescription drug claims, and enrollment information. Claims data in each of the subsections contain a unique patient identifier and information on patient age, gender, geographic location (including state and three-digit zip code), and type of health plan.

The inpatient and outpatient services subsections of the MarketScan database contain information on all services performed in an inpatient or outpatient setting. These data include information on dates of services, the diagnoses associated with the claim, and the procedures performed. The outpatient services subsection includes information for all services performed in a doctor’s office, hospital outpatient clinic, emergency room, or other outpatient facility. Previous studies have used the MarketScan database to examine health-care service use and costs for children (24–29).

### Study Population

The study sample consisted of children aged 6–15 with a diagnosis of one of the following conditions (ICD-9 codes in parentheses): OCD (300.3), AN (307.1), anxiety disorder (300.0–300.2), tic disorder (307.20 or 307.22), ADHD (314), major depression (296.2–296.3), and bipolar disorder (296.0–296.2, 296.4–296.8). To test the specificity of the models, we also included children with broken bones (800–829) and open wounds (870–897). To identify new cases, we further limited the sample in each diagnostic group to children who were continuously enrolled for at least 1 year prior to their first diagnosis for the condition (the index date). Next, a matched one-to-one control group was constructed for each diagnostic group consisting of children who did not have the condition of interest and were matched with their corresponding case on age, gender, date of the start of continuous enrollment, and three-digit zip code. Because vaccines tend to occur during certain times of year (such as before summer camps or the beginning of school), controls were also required to have an outpatient visit at which they did not receive a vaccine within 15 days of the date that the corresponding case was first diagnosed with the condition, in an effort to control for seasonality. The date of this visit was the index date for children in the control group.

For each diagnostic group and their corresponding controls, individuals who were vaccinated in the 3, 6, or 12 months before the index date were identified. Exposure to vaccines was measured using CPT codes (list available from the authors upon request) and ICD-9 codes (V03–V06 or V07.2). Exposure to specific vaccines, including influenza, tetanus and diphtheria (TD), hepatitis A, hepatitis B, meningitis, and varicella, was tracked.

### Statistical Analysis

The analyses were performed for each diagnostic group (and their controls) separately. Children with multiple conditions (e.g., ADHD and tic disorder) were included in each of the corresponding analytic groups. First, the proportion of children who were exposed to vaccines in the period before the index date was compared across the case and control groups. Next, bivariate conditional logistic regression models were estimated to determine the hazard ratios (HRs) and 95% confidence intervals (95% CIs) associated with the effect of vaccine exposure on having the condition of interest. Separate models were run for the 3-, 6-, and 12-month periods preceding the index date for each diagnostic group. The study was approved by the Penn State College of Medicine Institutional Review Board.

## Results

Characteristics of each of the diagnostic groups are presented in Table 1. Sample sizes ranged from 551 children diagnosed with AN to 85,151 children with a broken bone. The average age ranged from 9.5 ± 2.5 for children with tic disorder to 13.3 ± 1.7 for children with AN. Not surprisingly, the distribution of sex varied considerably across diagnostic groups, with higher percentages of females in the AN (86.6%) and major depression (56.3%) categories and higher proportions of males in the tic disorder (76.4%), ADHD (66.8%), open wound (62.2%), broken bone (58.4%), OCD (56.6%), and bipolar disorder (54.1%) categories.

**Table 1:** **Characteristics of the sample**.

Characteristic	Broken bone		Open wound		OCD		Anorexia nervosa		Anxiety disorder		Tic disorder		ADHD		Major depression		Bipolar disorder	
								
	*N*	%	*N*	%	*N*	%	*N*	%	*N*	%	*N*	%	*N*	%	*N*	%	*N*	%
*N*	85,151		73,290		3,222		551		23,462		2,547		46,640		13,295		5,892	
Age, mean ± SD	11.1 ± 2.7		10.6 ± 2.9		11.1 ± 2.6		13.3 ± 1.7		11.3 ± 2.8		9.5 ± 2.5		10.3 ± 2.8		12.9 ± 2.2		12.3 ± 2.6	
**Gender**																		
Male	49,689	58.4	45,562	62.2	1,825	56.6	74	13.4	11,357	48.4	1,945	76.4	31,170	66.8	5,811	43.7	3,185	54.1
Female	35,462	41.6	27,728	37.8	1,397	43.4	477	86.6	12,105	51.6	602	23.6	15,470	33.2	7,484	56.3	2,707	45.9
**Receipt of vaccine^a^**																		
Any vaccine	10,308	12.1	7,577	10.3	512	15.9	118	21.4	3,389	14.4	402	15.8	5,536	11.9	1,700	12.8	682	11.6
Influenza	3,550	4.2	2,783	3.8	246	7.6	42	7.6	1,418	6.0	214	8.4	2,366	5.1	548	4.1	247	4.2
TD	2,061	2.4	1,358	1.9	68	2.1	27	4.9	520	2.2	53	2.1	913	2.0	405	3.0	159	2.7
HepA	1,950	2.3	1,462	2.0	86	2.7	14	2.5	570	2.4	62	2.4	1,005	2.2	285	2.1	100	1.7
HepB	713	0.8	550	0.8	20	0.6	12	2.2	201	0.9	14	0.5	366	0.8	150	1.1	60	1.0
Meningitis	1,325	1.6	835	1.1	61	1.9	24	4.4	422	1.8	36	1.4	495	1.1	223	1.7	88	1.5
Varicella	1,002	1.2	750	1.0	52	1.6	8	1.5	323	1.4	44	1.7	577	1.2	93	0.7	42	0.7

*^a^Reciept of vaccine in the 6 months before first diagnosis of the disorder*.

*OCD, obsessive–compulsive disorder; ADHD, attention deficit hyperactivity disorder; TD, tetanus and diphtheria; Hep, hepatitis*.

Rates of receipt of vaccines in the 6 months before the first diagnosis of the disorder are also reported in Table 1 and varied considerably across diagnostic groups. Receipt of any vaccine in the previous 6 months was highest for children with AN (21.4%), followed by OCD (15.9%) and tic disorder (15.8%), and was lowest for children with open wounds (10.3%). Rates of receipt of specific vaccines were fairly low, ranging from 0.5% for the hepatitis vaccine among children with tic disorder to 8.4% for the influenza vaccine among children with tic disorder. In general, vaccination rates were highest among children in the AN, OCD, and tic disorder groups and were lowest for children in the open wound or bipolar disorder groups.

Table 2 presents HRs from the bivariate associations of receipt of vaccine within the 3-, 6-, and 12-month periods preceding the index date for each diagnostic group compared to their matched controls. Children with OCD, AN, anxiety disorder, or ADHD were more likely to have had a vaccination in each of the preceding periods than their matched controls, and children with tic disorder were more likely to have had a vaccination in the preceding 6- and 12-month periods than their matched controls. HRs associated with receipt of any vaccine were highest for children with AN, ranging from 1.47 (95% CI 1.12–1.93) for the 12-month preceding period to 1.80 (95% CI 1.21–2.68) for the 3-month preceding period, followed by OCD, which ranged from 1.23 for both the 12-month (95% CI 1.12–1.93) and 3-month (95% CI 1.02–1.49) preceding periods to 1.27 (95% CI 1.10–1.47) for the 6-month preceding period. However, children with broken bones were also more likely to have had a vaccination in the preceding period, although the HRs were smaller, ranging from 1.04 (95% CI 1.00–1.08) for the 3-month preceding period to 1.08 (95% CI 1.05–1.11) for the 6-month preceding period. The other control condition, open wounds, showed no increased incidence following vaccinations. In addition, children with major depression were *less* likely to have had a vaccination in all 3 preceding periods, and children with bipolar disorder were also *less* likely to have had a vaccination in the 6- or 12-month preceding periods.

**Table 2:** **Bivariate associations of receipt of vaccine with new diagnosis**.^a^

Vaccine	Broken bone *N* = 85,151			Open wound *N* = 73,290			OCD *N* = 3,222			Anorexia nervosa *N* = 551			Anxiety disorder *N* = 23,462			Tic disorder *N* = 2,547			ADHD *N* = 46,640			Major depression *N* = 13,295			Bipolar disorder *N* = 5,892		
								
	Hazard ratio (HR)	95% CI		HR	95% CI		HR	95% CI		HR	95% CI		HR	95% CI		HR	95% CI		HR	95% CI		HR	95% CI		HR	95% CI	
**Any vaccine**																											
3 months	**1.04**	**1.00**	**1.08**	0.96	0.92	1.00	**1.23**	**1.02**	**1.49**	**1.80**	**1.21**	**2.68**	**1.12**	**1.04**	**1.20**	1.11	0.90	1.38	**1.06**	**1.00**	**1.12**	**0.88**	**0.80**	**0.97**	0.87	0.75	1.01
6 months	**1.08**	**1.05**	**1.11**	0.97	0.94	1.01	**1.27**	**1.10**	**1.47**	**1.63**	**1.17**	**2.27**	**1.13**	**1.07**	**1.19**	**1.25**	**1.06**	**1.47**	**1.04**	**1.00**	**1.09**	**0.92**	**0.86**	**0.99**	**0.82**	**0.73**	**0.91**
12 months	**1.07**	**1.04**	**1.09**	**0.97**	**0.94**	**1.00**	**1.23**	**1.09**	**1.38**	**1.47**	**1.12**	**1.93**	**1.14**	**1.09**	**1.19**	**1.19**	**1.04**	**1.36**	**1.08**	**1.05**	**1.12**	**0.89**	**0.84**	**0.95**	**0.87**	**0.79**	**0.95**
**Influenza**																											
3 months	1.03	0.96	1.11	0.93	0.86	1.01	**1.36**	**1.02**	**1.82**	**2.20**	**1.10**	**4.38**	**1.23**	**1.10**	**1.38**	1.24	0.91	1.67	0.98	0.91	1.07	**0.81**	**0.68**	**0.96**	**0.71**	**0.55**	**0.92**
6 months	**1.07**	**1.02**	**1.13**	0.96	0.91	1.02	**1.48**	**1.21**	**1.83**	**1.83**	**1.07**	**3.15**	**1.24**	**1.14**	**1.35**	**1.27**	**1.02**	**1.58**	0.97	0.91	1.02	0.89	0.79	1.00	**0.84**	**0.70**	**1.00**
12 months	**1.06**	**1.02**	**1.09**	0.97	0.93	1.01	**1.35**	**1.16**	**1.59**	1.52	0.99	2.34	**1.27**	**1.19**	**1.35**	**1.28**	**1.08**	**1.50**	1.04	0.99	1.09	0.93	0.84	1.02	0.87	0.76	1.00
**TD**																											
3 months	1.02	0.94	1.11	0.92	0.83	1.02	1.15	0.72	1.84	1.70	0.78	3.71	0.95	0.80	1.13	0.86	0.47	1.60	1.04	0.91	1.19	0.95	0.78	1.16	0.83	0.62	1.12
6 months	1.07	1.00	1.14	0.94	0.87	1.01	1.07	0.75	1.51	1.77	0.90	3.49	0.91	0.80	1.03	1.24	0.82	1.88	1.03	0.93	1.13	0.96	0.83	1.10	0.82	0.66	1.02
12 months	**1.07**	**1.02**	**1.12**	**0.93**	**0.88**	**0.98**	0.99	0.77	1.26	**1.63**	**1.05**	**2.52**	0.98	0.90	1.07	0.93	0.67	1.30	1.04	0.97	1.11	0.90	0.82	1.00	**0.80**	**0.68**	**0.93**
**HepA**																											
3 months	1.02	0.94	1.12	0.97	0.88	1.07	1.47	0.92	2.33	1.60	0.52	4.89	1.00	0.85	1.18	1.13	0.70	1.83	1.03	0.92	1.17	0.86	0.68	1.08	1.03	0.73	1.47
6 months	1.05	0.98	1.12	0.99	0.92	1.07	**1.43**	**1.02**	**2.01**	1.09	0.48	2.51	1.08	0.95	1.22	1.35	0.92	1.98	1.05	0.96	1.15	0.95	0.81	1.13	0.79	0.60	1.03
12 months	**1.08**	**1.02**	**1.13**	0.99	0.93	1.05	**1.40**	**1.07**	**1.82**	1.73	0.89	3.37	1.00	0.91	1.10	1.17	0.88	1.56	**1.09**	**1.02**	**1.18**	0.97	0.86	1.11	0.81	0.66	1.00
**HepB**																											
3 months	1.08	0.93	1.25	1.05	0.89	1.24	0.71	0.32	1.61	3.00	0.61	14.86	1.01	0.76	1.34	1.40	0.44	4.41	1.13	0.91	1.39	1.05	0.77	1.43	0.97	0.61	1.56
6 months	1.02	0.92	1.13	1.02	0.91	1.15	0.80	0.45	1.44	1.71	0.68	4.35	1.01	0.83	1.23	1.17	0.54	2.52	1.07	0.92	1.24	0.89	0.71	1.11	0.91	0.64	1.29
12 months	1.03	0.95	1.11	1.00	0.91	1.09	0.93	0.60	1.44	1.55	0.72	3.30	1.01	0.89	1.16	1.19	0.61	2.31	1.06	0.95	1.18	1.00	0.85	1.18	1.07	0.83	1.38
**Meningitis**																											
3 months	1.05	0.95	1.17	1.04	0.92	1.19	1.10	0.67	1.80	1.71	0.68	4.35	1.06	0.88	1.27	1.46	0.72	2.95	1.16	0.98	1.38	0.89	0.70	1.13	0.87	0.57	1.33
6 months	1.08	1.00	1.17	1.02	0.92	1.12	1.15	0.78	1.71	1.75	0.86	3.56	1.12	0.97	1.29	**1.94**	**1.08**	**3.46**	1.08	0.95	1.23	0.88	0.73	1.05	0.82	0.61	1.11
12 months	1.06	0.99	1.14	1.02	0.94	1.10	1.34	0.96	1.87	1.42	0.79	2.56	**1.14**	**1.01**	**1.29**	**1.73**	**1.07**	**2.80**	1.06	0.95	1.18	**0.81**	**0.70**	**0.94**	0.85	0.67	1.09
**Varicella**																											
3 months	**0.88**	**0.79**	**0.99**	0.90	0.79	1.03	1.33	0.79	2.26	1.00	0.20	4.96	1.06	0.85	1.31	0.73	0.42	1.27	1.06	0.90	1.24	0.85	0.58	1.24	1.08	0.63	1.87
6 months	0.97	0.88	1.06	0.96	0.87	1.07	1.38	0.89	2.15	2.66	0.71	10.04	1.17	0.99	1.38	0.91	0.59	1.40	1.09	0.97	1.23	0.85	0.64	1.14	0.79	0.52	1.21
12 months	1.00	0.93	1.08	0.93	0.85	1.01	1.36	0.92	1.99	1.29	0.48	3.45	1.11	0.97	1.28	0.97	0.67	1.40	1.06	0.95	1.17	0.84	0.66	1.06	0.74	0.53	1.05

*^a^Cases and controls matched on date (±15 days, see text) of the start of continuous enrollment, year of birth, gender, and three-digit zip code. *N*’s represent cases only. Results in bold are statistically significant at *p* < 0.05*.

*OCD, obsessive–compulsive disorder; ADHD, attention deficit hyperactivity disorder; TD, tetanus and diphtheria; Hep, hepatitis*.

There were fewer statistically significant results when looking at the effects of the individual vaccines. Children with OCD were more likely to have received the influenza vaccine in each of the preceding periods, or the hepatitis A vaccine in the previous 6 or 12 months. Children with AN were also more likely to have received the influenza vaccine in the preceding 3 or 6 months, or the TD vaccine in the previous 12 months. Children with anxiety disorder were more likely to have received the influenza vaccine in the previous 12 months. Children with tic disorder were more likely to have received an influenza or a meningococcal vaccine in the previous 6 or 12 months. However, children with broken bones were also slightly more likely to have received the influenza vaccine during the previous 3-, 6-, and 12-month intervals. In contrast, children with major depression were *less* likely to have received the influenza vaccine in the previous 3 months or the meningitis vaccine in the previous 12 months. Similarly, children with bipolar disorder were also *less* likely to have received the influenza vaccine in the previous 3 or 6 months. Antecedent vaccination with any vaccine and with the TD vaccine during the previous 12 months was very modestly associated with a *decreased* incidence of open wounds (Table 2).

## Discussion

The principal findings of this study are as follows: (i) children with OCD, AN, anxiety disorder, and tic disorder were more likely to have received influenza vaccine during the preceding 1-year period (for OCD in the preceding 3-, 6-, and 12-month periods; for AN in the preceding 3- and 6-month periods; for anxiety disorder in the preceding 6- and 12-month periods; for tic disorder in the preceding 6- and 12-month periods) and (ii) HRs associated with receipt of any vaccine were highest for children with AN, ranging from 1.47 for the 12-month preceding period to 1.80 for the 3-month preceding period, followed by OCD, which ranged from 1.23 for both the 12- and 3-month preceding periods to 1.27 for the 6-month preceding period. However, if we apply a high standard [so that the upper limit of the of the 95% CI of the HR observed for the association between the administration of any vaccine and the subsequent occurrence of a broken bone (1.11) falls below the lower limit of the 95% CI observed for any of the HRs for any of the neuropsychiatric disorders], only the findings for AN pass this stringent threshold (Table 2). Applying a similar high standard for the individual vaccines, the only associations that pass this threshold concern the influenza vaccine given in the preceding 6- and 12-month periods for OCD and anxiety disorders.

Our findings showing that children with AN, OCD, or a tic disorder were more likely to have received the influenza vaccine in the preceding periods were noteworthy given the findings of increased incidence of narcolepsy in Finland, Sweden, Ireland, Norway, England, and France after vaccination with AS03-adjuvanted H1N1 vaccine (17, 18). Studies also show a threefold increase in the incidence of narcolepsy after following the 2009 H1N1 pandemic in China (30). Although the strong association between HLA class II and narcolepsy suggests that narcolepsy may be an autoimmune disorder, the exact mechanism leading to immune-related narcolepsy is not completely understood and other host factors are likely to play an important role (31, 32). Investigators have made use of *in silico* techniques to begin to identify potential causal pathways and the relevant host factors (19). More recently, Ahmed et al. (23) have shown that the H1N1 influenza vaccine containing the AS03 adjuvant triggers antibodies that bind to hypocretin receptor 2a. Additional work is needed to replicate and extend these findings.

It is also of note that the observed association between the antecedent administration of the influenza vaccine and the new onset of AN and OCD may suggest that aberrant immune functioning may be a common pathogenetic pathway for OCD and AN. The high comorbidity rates between OCD and AN, common cortico-striatal abnormalities in neuroimaging studies, and anti-putamen antibodies both in OCD and AN cases are some of the shared features of these two disorders worth considering (33–35). In addition, the increased risk for autoimmune disorders (such as type 1 diabetes mellitus, Crohn’s disease, and celiac disease) in eating disorders (36) and the documented comorbidity of OCD and autoimmune diseases (such as systemic lupus erythematosus, thyroid dysfunction, and multiple sclerosis) (35) indicate the possible shared host factors and the role of immune-mediated mechanisms in the development of AN and OCD. We also note the findings of Zastrow and colleagues that vaccination to prevent H1N1 influenza is recommendable even in extremely underweight AN patients (37).

Limitations of this study include that we were unable to control for the fact that providers may designate ICD-9 insurance billing codes for vaccines generally without specifying the particular vaccine. Additionally, we were unable to match claims by providers in order to control for the diagnostic predilections of individual physicians and account for the possibility that some physicians might be more (or less) likely to diagnose one or more neuropsychiatric disorders and/or recommend specific vaccinations. The results of this study are further qualified by the limitations of the administrative retrospective data used in this study, rather than from systematically obtained clinical data, especially around diagnostic classification. This is a shortcoming inherent in studies that rely on secondary analyses to secure large sample sizes. Furthermore, early vaccines are grouped together in the first 15 months of infancy, some of them given simultaneously at one visit and received by most of the infants. This leads to a limitation in analyzing the possibility of the temporal association between individual vaccines and the onset of neuropsychiatric disorders. We deliberately chose our sample from children aged 6–15 years in order to overcome this limitation. Another limitation concerns changes in vaccine guidelines during the time interval used in this analysis. For example, the American Academy of Pediatrics first recommended the use of the conjugate meningococcal vaccine in August 2005 and the varicella booster in April 2007. As a consequence, the size of the cohorts who received these vaccines is smaller in comparison to other vaccines. Another issue concerns the fact that the influenza vaccination is an annual vaccination using a vaccine specific for a given year to protect against the highly variable influenza virus. As a consequence, it is also the most frequently administered vaccine that indeed may well have disproportionately “driven” the “any vaccine” findings (Table 1). Given its variability and prevalence, in future studies, it will be important to look year-by-year. Perhaps the largest limitation and potential threat to the study’s validity has to do with the fundamental impossibility of detecting a causal relationship within the context of such a case–control study. Indeed, this provides no more than a relative perspective of the potential risk, as opposed to the absolute risk (the real proportion of individuals who had a vaccination and then developed one or more of the investigated conditions) that might be expected to be reasonably small.

Moving forward, our findings require replication in a larger population-based sample, possibly including assessments of various potentially important host factors, e.g., the individual’s genomic and epigenomic background, the individual’s microbiome, their history of antecedent psychosocial stress, infections, as well as other potentially simultaneously administered vaccinations, the differences in vaccine types, and the route of administration (e.g., intramuscular or intranasal administration of influenza vaccine) as different routes of administration may lead to a difference in immune responses in the host.

It will also be critically important to determine whether or not newly acquired infectious diseases against which the children were vaccinated may themselves lead to an increased incidence to one or more of these neuropsychiatric disorders. In fact, it would not be surprising if the diseases *per se* represent a stronger risk factor than vaccinations. The documented increase in the incidence of narcolepsy following the 2009 H1N1 pandemic in China provides a clear example (30). Our earlier epidemiological study documenting a temporally related modest increase in the incidence of OCD, tic disorders, and ADHD following a prior streptococcal infection provides another example (25). Future epidemiologic investigations are needed to address this important question.

The present study has the potential to extend our knowledge about the role of the immune system in some pediatric-onset neuropsychiatric disorders. However, our findings do not demonstrate a causal role of vaccination in the pathoetiology of any of these conditions. This is especially important given the clear public health benefits of the timely administration of vaccines in preventing mortality and morbidity (38). Vaccines are among the most successful and cost-effective preventive public health interventions (39). Vaccination has led to eradication of smallpox, and we are close to the eradication of poliomyelitis across the world. Since most of the vaccine-preventable diseases are contagious from person to person, the increase in numbers of vaccinated individuals will decrease the chance of a disease to spread. Proper vaccination not only protects our generation but also protects future generations against epidemics of diseases. It should always be kept in mind that vaccines are crucial for eradicating infectious diseases and preventing the higher rates of morbidity and mortality due to infections. However, care should be taken to ensure that children scheduled to receive vaccinations are in good health and that recommended precautions are taken at the time a vaccine is to be administered.

### Clinical Significance

These findings provide preliminary epidemiologic evidence that the onset of some pediatric-onset neuropsychiatric disorders, including AN, OCD, anxiety disorders, and tic disorders, may be temporally related to prior vaccinations. Each of these conditions is etiologically heterogeneous, and host factors likely play an important role in a small subset of vulnerable individuals. However, these findings, even if replicated in future studies, do not prove a causal role of vaccination in the pathoetiology of any of these conditions. Indeed, antecedent infections may also increase the risk of developing one or more of these disorders in vulnerable individuals. Finally, given the modest magnitude of these findings and the clear public health benefits of the timely administration of vaccines in preventing mortality and morbidity in childhood, we encourage families to maintain the currently recommended vaccination schedules while taking all necessary precautions as documented by the Centers for Disease Control and Prevention (http://www.cdc.gov/vaccines/recs/vac-admin/contraindications.htm).

## Author Notes

Data were obtained from the MarketScan^®^ Commercial Claims and Encounters database, which is constructed and maintained by Truven Health Analytics.

## Author Contributions

DL, RK, BR, and JL designed the study and wrote the protocol. SG commented on the protocol. DL undertook the statistical analysis. BR, DL, and JL wrote the first draft of the manuscript. All the authors commented on the manuscript. All the authors contributed to and have approved the final manuscript.

## Conflict of Interest Statement

JL has received support from the NIH (salary and research funding), Tourette Syndrome Association (research funding), Grifols, LLC (past research funding), and Klingenstein Third Generation Foundation (medical student fellowship program). He receives book royalties from John Wiley and Sons, McGraw Hill, and Oxford University Press. The other authors declare no conflict of interest. The reviewer ML and handling Editor declared their shared affiliation, and the handling Editor states that the process nevertheless met the standards of a fair and objective review.
